# The Development of a Juvenile Porcine Augmented Renal Clearance Model Through Continuous Infusion of Lipopolysaccharides: An Exploratory Study

**DOI:** 10.3389/fvets.2021.639771

**Published:** 2021-04-29

**Authors:** Laura Dhondt, Siska Croubels, Robin Temmerman, Pieter De Cock, Evelyne Meyer, Wim Van Den Broeck, Peter De Paepe, Mathias Devreese

**Affiliations:** ^1^Department of Pharmacology, Toxicology and Biochemistry, Ghent University, Ghent, Belgium; ^2^Department of Pharmacy, Ghent University Hospital, Ghent, Belgium; ^3^Heymans Institute of Pharmacology, Ghent University, Ghent, Belgium; ^4^Department of Paediatric Intensive Care, Ghent University Hospital, Ghent, Belgium; ^5^Department of Morphology, Faculty of Veterinary Medicine, Ghent University, Ghent, Belgium

**Keywords:** piglet, lipopolysaccharides, sepsis animal model, augmented renal clearance, continuous infusion

## Abstract

Augmented renal clearance (ARC) as observed in the critically ill (pediatric) population can have a major impact on the pharmacokinetics and posology of renally excreted drugs. Although sepsis has been described as a major trigger in the development of ARC in human critically ill patients, mechanistic insights on ARC are currently lacking. An appropriate ARC animal model could contribute to reveal these underlying mechanisms. In this exploratory study, a state of ARC was induced in 8-week-old piglets. Conscious piglets were continuously infused over 36 h with lipopolysaccharides (LPS) from *Escherichia coli* (O111:B4) to induce sepsis and subsequently trigger ARC. To study the dose-dependent effect of LPS on the renal function, three different doses (0.75, 2.0, 5.0 μg/kg/h) were administered (two ♂ piglets/dose, one sham piglet), in combination with fluid administration (0.9% NaCl) at 6 ml/kg/h. Single boluses of renal markers, i.e., creatinine [40 mg/kg body weight (BW)], iohexol (64.7 mg/kg BW), and para-aminohippuric acid (PAH, 10 mg/kg BW) were administered intravenously to evaluate the effect of LPS on the renal function. Clinical parameters were monitored periodically. Blood sampling was performed to determine the effect on hematology, neutrophil gelatinase-associated lipocalin, and prostaglandin E_2_ plasma levels. All piglets that were continuously infused with LPS displayed an elevated body temperature, heart rhythm, and respiratory rate ~1–3 h after start of the infusion. After infusion, considerably higher total body clearances of iohexol, creatinine, and PAH were observed, independent of the administration of LPS and/or its dose. Since also the sham piglet, receiving no LPS, demonstrated a comparable increase in renal function, the contribution of fluid administration to the development of ARC should be further evaluated.

## Introduction

Sepsis and septic shock are both significant causes of morbidity and mortality in the intensive care population. The incidence of sepsis, based on data of high-income-countries, is estimated at 31.5 million cases per year, with potentially 5.3 million deaths annually (1). Furthermore, it is assumed that the incidence of sepsis is the highest in neonates and children (2). In septic patients, important pathophysiological changes may occur as a consequence of the underlying disease state and medical interventions (3–6). One of the organs frequently affected is the kidney.

Deterioration of the kidney function as result of acute kidney injury is frequently reported as a serious complication of sepsis. Currently, renal dose adjustments have only been recommended in patients with impaired renal function because these patients may be at risk for drug toxicity (7). Recently, the phenomenon of augmented renal clearance (ARC) has increasingly been recognized in subsets of critically ill (pediatric) patients, with sepsis being suggested as one of the main triggers (4, 7–11). These patients display a considerably enhanced drug excretion, potentially leading to subtherapeutic concentrations. Therefore, increased dosages may be necessary to maintain therapeutic concentrations of various renally excreted drugs. This is particularly relevant for renally excreted antibiotics in critically ill patients, since detrimental effects of altered drug disposition on morbidity and mortality have been suggested (9). In addition, these subtherapeutic plasma concentrations may predispose to emergence of resistant target pathogens (4, 7–11). Although sepsis has been described as a trigger in the development of ARC, profound insights on the underlying mechanisms of ARC are currently lacking. An appropriate ARC animal model that could be obtained through the induction of sepsis would contribute to reveal these underlying mechanisms.

Sepsis animal models can be generally classified based upon the type of initiating agent [lipopolysaccharides (LPS), live bacteria, fecal content] and the species in which sepsis is induced. The species is an important variable to consider (12). Small laboratory animal species, such as rats, mice, and guinea pigs, are frequently used for survival studies or when a large number of animals is required, mainly due to their low cost. On the other hand, sheep is frequently used in chronic, unanesthetized models (12). The pig has become important as experimental large animal species over the last years (12). Pigs closely resemble humans with respect to size, anatomy, and physiology of the kidneys and other organ systems (cardiovascular, gastrointestinal tract, and liver) (13). Furthermore, it has been demonstrated that mature renal values of the glomerular filtration rate (GFR), effective renal plasma flow (ERPF), anion secretion, and tubular reabsorption were in the same order of magnitude in humans and pigs (14). Therefore, pigs can provide a more accurate and reliable prediction of human pharmacokinetic (PK) behavior of drugs compared with classically used rodent and even to other non-rodent species (13, 15). In addition, as the costs associated with conventional pigs are relatively low, they are increasingly used for physiological and pharmacological studies, including experiments in the area of sepsis and septic shock (12, 16).

A sepsis-like state can be obtained by the injection of an exogenous (endo) toxin. Endotoxins, such as LPS, are components on the outer membrane of the cell wall of Gram-negative bacteria and are involved in the pathogenesis of sepsis. Since the LPS model is simple, sterile, and partly resembles with human sepsis pathophysiology, the LPS infusion/injection model has been widely used for sepsis research. Nevertheless, large differences are present within the published endotoxicosis models. This can partly be explained by the variation in administration route. LPS can be administered either intraperitoneally (17, 18), or intravenously (IV) either as bolus injection (19, 20), or via continuous infusion (21, 22). In contrast to a single bolus IV injection, a continuous LPS infusion is considered to mimic clinical endotoxemia/septicemia more accurately because endotoxins are released into the circulation over an extended period of time (16). Large variations are also present in the reported type and dose of LPS, resulting in important differences in host response. In dogs, the hemodynamic response observed in the case of a very low-dose continuous IV infusion of LPS is quite different from a high-dose IV bolus of LPS (16, 23). A third relevant difference between these preclinical LPS-induced sepsis models is the extent to which supportive therapy is applied. Intravascular volume resuscitation, intubation, mechanical ventilation, and inotropic agent infusion are different techniques often used in the intensive care unit (ICU) to increase the survival rate of the critically ill patient. Some of the published models do not use any of these supportive therapies, possibly making them less relevant as a model for the human septic critically ill population (24).

The main objective of the current explorative study was to develop a juvenile, porcine ARC model by inducing sterile sepsis. For this purpose, LPS was IV administered to 8-week-old, conscious piglets over a 36-h period. Juvenile pigs were used because the increasing evidence that ARC is highly prevalent in the critically ill pediatric population (25). Three different LPS doses (i.e., 0.75, 2.0, and 5.0 μg/kg/h) were selected to evaluate possible dose-dependent responses. Subsequently, the effect of this continuous infusion on the renal function and hematology parameters was established. Renal effects of LPS were evaluated using the GFR markers, iohexol, and exogenously administered creatinine, and the ERPF marker, para-aminohippuric acid (PAH). Furthermore, neutrophil gelatinase-associated lipocalin (NGAL, lipocalin-2) was used as biomarker for sepsis-induced AKI (26–28). In addition, renal morphological changes were investigated. Since prostaglandins play an important role in the control of renal blood flow, febrile response and increased PGE_2_ levels have been observed in septic patients, and plasma PGE_2_ levels were additionally quantified in the studied pigs (29–31). As fluids are often administered as supportive therapy in septic (pediatric) patients and may contribute to the development of ARC, a constant rate infusion (CRI) of 0.9% NaCl solution at a rate of 6 ml/kg/h was given simultaneously with the LPS administration (32, 33).

## Materials and Methods

### Animals

The current study was conducted with consent of the Ethical Committee of the Faculty of Veterinary Medicine and the Faculty of Bioscience Engineering of Ghent University (EC 2017/24). Care and use of animals were in full compliance with the Belgian (Belgian Royal Decree of May 29, 2013) and European legislation on animal welfare and ethics (2010/63/EU) (34, 35).

Seven, 8-week-old, male piglets (Landrace x large white, Seghers Hybrid®, RA-SE Genetics, Belgium) were purchased. Upon arrival, the piglets were group housed in standard pig stables (2.30 × 2.40 m), where *ad libitum* water and feed (Piggistart Opti®, Aveve, Leuven, Belgium) were provided. Stable temperature was on average 23.2 ± 0.45°C. During the whole experiment, the stables were enriched with rubber toys and cotton towels. After an acclimatization period of 3 days, a double lumen catheter (two-lumen central venous catheterization set, 7 Fr, 60 cm; Arrow® International) was surgically placed in the external jugular vein following the procedure described by Gasthuys et al. (36, 37). After surgery, the piglets were housed individually to avoid displacements of the catheters. Catheters were flushed twice daily with heparinized 0.9% NaCl solution (50 IU/ml), and the bandages were changed daily.

### Experimental Design

A graphical illustration of the trial design is shown in Supplementary Figure 1. Following surgery, the piglets could recover for 1 day. The next day, the following renal markers were administered IV to all piglets [18.47 ± 1.96 kg body weight (BW)] to obtain baseline measurements of the renal function: iohexol [64.7 mg/kg BW, Omnipaque 300® (GE Healthcare, Eindhoven, The Netherlands)], PAH (10 mg/kg BW), and creatinine (40 mg/kg BW). The commercially available powders of creatinine hydrochloride and sodium PAH, both purchased from Sigma-Aldrich (Bornem, Belgium), were dissolved simultaneously in a 0.9% NaCl solution at a final concentration of 0.14 g/ml creatinine and 0.035 g/ml PAH. Iohexol and the PAH-creatinine solution were consecutively administered as bolus injection through the proximal lumen of the jugular catheter. Subsequently, venous blood samples (1.5 ml) were collected from the distal lumen into K_3_-EDTA collection tubes (Vacutest Kima®, Piove di Sacco, Italy) at 0 (prior to administration), 5, 15, 30, 45, 60 min and 2, 3, 6, 8, and 12 h post-administration and immediately kept on ice. Samples were centrifuged (2,095 × *g*, 10 min, 4°C) within 2 h of sampling, and aliquots of plasma were stored at ≤-15°C until analysis.

After 1 day of recuperation period, the piglets (19.54 ± 1.75 kg) were continuously infused over 36 h with ultrapure LPS from *Escherichia coli* (O111:B4) (InVivogen, Toulouse, France). Six piglets were randomly assigned to one of the three different LPS doses (0.75; 2.0; 5.0 μg/kg/h), which were IV administered through the proximal catheter (two piglets/dose). The LPS was dissolved in 0.9% NaCl solution. The final concentration of the LPS solution was as such that each piglet received concomitantly 0.9% NaCl solution at 6 ml/kg/h as fluid therapy. One piglet was included as sham animal and received only fluid treatment. The animals' clinical condition was scored continuously by qualified personnel observing symptoms and behavior during the LPS challenge. More specifically, the occurrence of nausea, vomiting, shivering, and lateral/sternal decubitus was registered. Body temperature was measured by a Lifechip Biothermo (Allflex,Vitré, France), placed in the *musculus gluteus maximus* during surgery. Also heart rhythm and respiration frequency were recorded without fixating the piglets. These measurements were performed hourly on the first 4 h of the LPS infusion, followed by two hourly measurements for the remaining part of the experiment.

Blood samples (1 ml) for PGE_2_ analysis were collected into heparin- (10 IU; Leo Pharma) and indomethacin-coated (10 μg/ml) microcentrifuge tubes, before (0 h) and at 0.5, 1, 2, 3, 4, 6, 8, 12, 18, 24, 30, and 36 h after the start of the LPS infusion. Indomethacin was added to prevent *ex vivo* artifactual eicosanoid generation (38). Samples were immediately placed on ice and centrifuged (3,500 × *g*, 10 min, 4°C) within 2 h. Plasma was isolated, aliquoted, and stored at ≤-70°C until analysis.

In order to quantify the effect on the renal function, the abovementioned renal markers were administered IV at 4 and 24 h after the start of LPS infusion. Each time, 1.5 ml-blood samples were collected at the same time points as described for the baseline measurement. Additionally, a 1.5 ml-blood sample before and at 12, 24, and 36 h of LPS administration was taken to evaluate the changes in white blood cell count and formula. These measurements were performed by Medvet BVBA (Antwerp, Belgium).

To quantify plasma NGAL (pNGAL) levels, additional blood sampling (1 ml) was performed just before and at 4 and 24 h after the start of the infusion. Samples were immediately placed on ice and centrifuged (2,095 × *g*, 10 min, 4°C) within 2 h of sampling. To evaluate urinary NGAL (uNGAL) levels, urine pouches were attached to the prepuce of the piglets 4 h after the start of the infusion following the method of Gasthuys et al. (37). Only the urine of the first voiding was subjected to NGAL analysis. Voided urine and plasma were aliquoted and stored at ≤-70°C until analysis.

After the 36 h of LPS administration, euthanasia was performed by administration of an overdose of sodium pentobarbital (Sodium pentobarbital 20%®, Kela, Hoogstraten, Belgium). Immediately after euthanasia, samples were taken from both kidneys for morphological evaluation.

### Processing of Renal Markers in Plasma and Pharmacokinetic Analysis

Plasma concentrations of iohexol, PAH, and creatinine were measured simultaneously using an in-house validated ultra-high performance liquid chromatography-tandem mass spectrometry method (UHPLC-MS/MS), as previously described by Dhondt et al. (39). The lower limit of quantification (LLOQ) in plasma was 0.25 μg/ml for iohexol and PAH, and a relative LLOQ of 60.14 ± 7.64% was obtained for creatinine. The relative LLOQ was calculated as the ratio of the lowest spiked creatinine concentration that could be measured with acceptable accuracy and precision and the basal/endogenous concentration of creatinine in the matrix.

Plasma concentration–time data of iohexol, PAH, and creatinine were analyzed using compartmental PK analysis using Phoenix® 8.1. software (Certara, USA). Plasma concentrations below LLOQ/rLLOQ were excluded from the analysis. The estimated primary parameters were volume of distribution (V_d_) and total body clearance (CL_TOT_). Since glomerular filtration rate (GFR) in humans is generally indexed by body surface area (BSA), the Meeh-equation (*BSA* (*dm*^2^) = 9 × *BW* (*kg*)^2/3^) was used to convert BW to BSA.

### Analysis of Prostaglandins in Plasma

PGE_2_ is rapidly converted to its major inactive metabolite 13,14-dihydro-15-keto PGE_2_ (PGEM). Subsequently, PGEM can be further metabolized to 13,14-dihydro-15-keto PGA_2_ (PGAM) and finally to bicyclo PGE_2_ (Supplementary Figure 2) (40, 41)_._ Therefore, both plasma concentrations of PGE_2_ and its metabolites PGEM and PGAM were quantified using an in-house validated UHPLC-MS/MS method (De Baere et al., unpublished data). In brief, a sample preparation consisted of a liquid–liquid extraction procedure using hexane:ethyl acetate (50:50; v/v) acidified with 25 μl of 1 N hydrochloric acid. After evaporation of the supernatant under a gentle nitrogen stream (40 ± 5°C), the dry residue was reconstituted in 125 μl of water/MeOH (90:10, v/v). A 10-μl aliquot of the final clear solution was injected onto the UHPLC-MS/MS instrument. The LLOQ was set at 25, 100, and 50 pg/ml for PGE_2_, PGEM, and PGAM, respectively. The limit of detection was established at 4.06, 30, and 18.5 pg/ml for PGE_2_, PGEM, and PGAM, respectively.

### Analysis of Neutrophil Gelatinase-Associated Lipocalin in Plasma and Urine

Plasma and uNGAL were quantified using a commercial enzyme-linked immunosorbent assay (Pig Lipocalin-2 ELISA-kit, Abcam, Cambridge, United Kingdom), according to the manufacturer's protocol. The intra- and interday variation for this assay was estimated to be below 4 and 10%, respectively.

### Morphological Analysis

Immediately after euthanasia, four tissue samples of the renal cortex of each kidney were taken. Tissue samples of 0.5 cm3 were fixed in phosphate-buffered saline containing 4% formaldehyde for 24 h and were subsequently dehydrated in graded concentrations of ethanol. Thereafter, samples were embedded in paraffin. Tissue sections of 5 μm were deparaffinized, rehydrated, and then stained with hematoxylin and eosin (H&E). The samples were evaluated using a BX61 Olympus microscope (Olympus Soft Imaging solutions GMBH, Munster, Germany). All histological examinations were performed in a blinded way. Every tissue slice was evaluated using a visual field enlargement of 200:1 to get a first overview and finally scored using a visual field enlargement of 400:1. In total, 32 cortical fields were evaluated for each piglet. The samples were evaluated on tubular injury, i.e., dilatation of the tubulus diameter/flattening of the tubular cells, cast formation, and tubular cell vacuolization/degradation.

### Statistical Analysis

The correlation between GFR values, calculated as iohexol and exogenous creatinine CL_TOT_, was determined by a Pearson correlation test after removing potential outliers. A Bland–Altman plot was used to measure bias over the range of measured GFR values by comparison of both clearances. To obtain such a plot, the % difference between the GFR values obtained by the two methods at baseline, and after 4 and 24 h of LPS infusion was plotted against the average of the GFR values. Due to the limited sample size, no further statistical analysis was performed. Where appropriate, mean ± standard deviation (SD) is presented.

## Results

### Body Temperature

**Figure 1:**
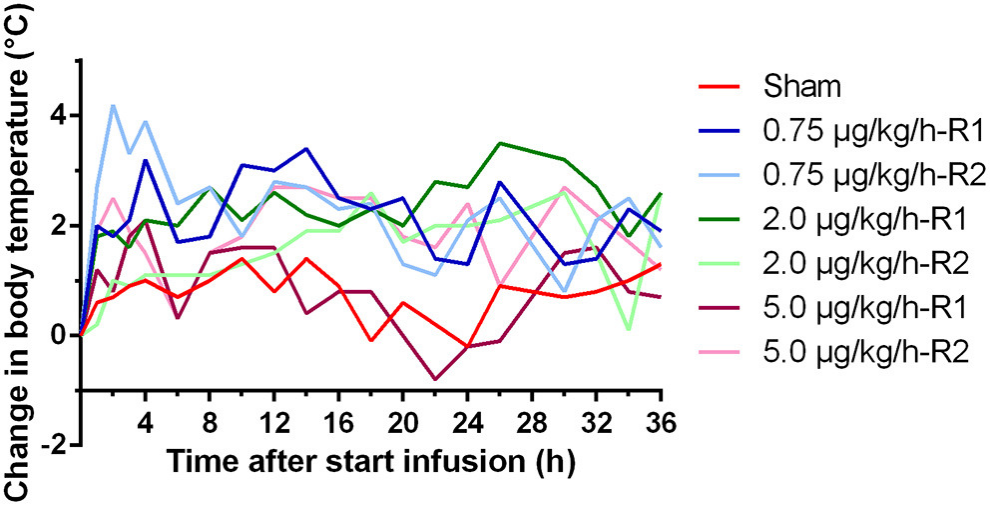
Change in body temperature over time during a 36 h continuous intravenous infusion of either 0.9% NaCl solution (6 ml/kg/h, sham, *n* = 1) or 0.9% NaCl solution (6 ml/kg/h) in combination with LPS (0.75; 2.0, and 5.0 μg/kg/h, *n* = 2/group). The change in body temperature is calculated as the difference between the body temperature at each time point and the temperature just before the start of the infusion. R1 and R2 present two replicates for each dose.

At the start of the experiment (time = 0 h), the mean (±SD) baseline body temperature (BT) of all piglets was 38.2 ± 0.98°C. After the start of the LPS administration, an increase in BT was observed in all LPS-challenged piglets. This elevated BT remained generally present during the whole duration of LPS infusion, as shown in Figure 1. The change in BT against the baseline measurement was provided instead of the absolute body temperatures to correct for the depth of the temperature chip as possible confounding factor. Maximal body temperatures were reached within a wide time interval of 2–30 h after the start of the LPS infusion and ranged between 39.9 and 42.2°C.

### Clinical Scoring

Before the start of the LPS challenge, all piglets were in good health, had a normal appetite, and displayed no adverse signs. Within 1 h after the start of the LPS administration, lethargy was observed in all LPS-receiving piglets but not in the sham piglet. Table 1 provides an overview of the clinical symptoms occurring during the LPS infusion.

**Table 1:** Overview of the clinical observations after administration of LPS (0.75; 2.0 or 5.0 μg/kg/h, *n* = 2/group) in combination with 6 ml/kg/h of 0.9% NaCl solution to 8-week-old piglets.

**Clinical symptoms**	**LPS-0.75 μg/kg/h (*n* = 2)**		**LPS-2.0 μg/kg/h (*n* = 2)**		**LPS-5.0 μg/kg/h (*n* = 2)**		**Sham (*n* = 1)**	
	**Prevalence**	**Time a.s.i. (h)**	**Prevalence**	**Time a.s.i. (h)**	**Prevalence**	**Time a.s.i. (h)**	**Prevalence**	**Time a.s.i. (h)**
**Tremor**	2/2	<1 h	2/2	1–2 h	2/2	1–2 h	0/1	N/A
**Lateral/sternal decubitus**	2/2	<2 h	2/2	<2 h	2/2	<2 h	0/1	N/A
**Lethargy**	2/2	<1 h	2/2	<1 h	2/2	<1 h	0/1	N/A
**Vomiting**	1/2	<1 h	1/2	2 h	1/2	1 h	0/1	N/A

*The sham piglet only received 0.9% NaCl solution. The time of the onset of the symptoms after start of infusion (a.s.i.) is also presented*.

*a.s.i., after start of the infusion; N/A, not applicable*.

**Figure 2:**
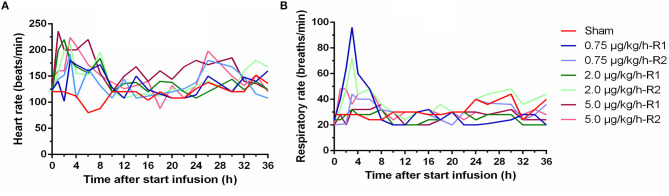
Time course of heart rate **(A)** and respiratory rate **(B)** during a 36 h continuous intravenous infusion of either 0.9% NaCl solution (6 ml/kg/h, sham, *n* = 1) or 0.9% NaCl solution (6 ml/kg/h) in combination with lipopolysaccharides (LPS) (0.75, 2.0, and 5.0 μg/kg/h, *n* = 2/group).

Between the animals receiving LPS, the severity of the symptoms seemed quite comparable. Lateral and sternal decubitus was generally observed within 2 h after start of the infusion (a.s.i.) and continued over the whole period of LPS administration. Anorexia, as demonstrated by a complete loss of interest in feed and drinking water, was observed in all piglets receiving LPS. This was probably attributed to the LPS induced nausea which resulted in vomiting in 50% of the piglets receiving LPS within 2 h a.s.i. In contrast to the other piglets in the LPS group, piglets receiving 0.75 μg/kg/h showed to a minimal extent a regained appetite and less lethargy at 8 h a.s.i. The latter manifested itself as occasional standing up. Tremor was observed in all LPS piglets, which was associated with the elevated BT and was present especially on the first hours of the LPS infusion. Both elevated heart rhythm and respiratory rate were typically noticed within 8 h a.s.i. but were not LPS dose dependent (Figure 2). In contrast, the sham piglet demonstrated normal behavior throughout the whole experiment, which manifested itself in playing, eating, social interaction, and sleeping.

### Hematology

**Figure 3:**
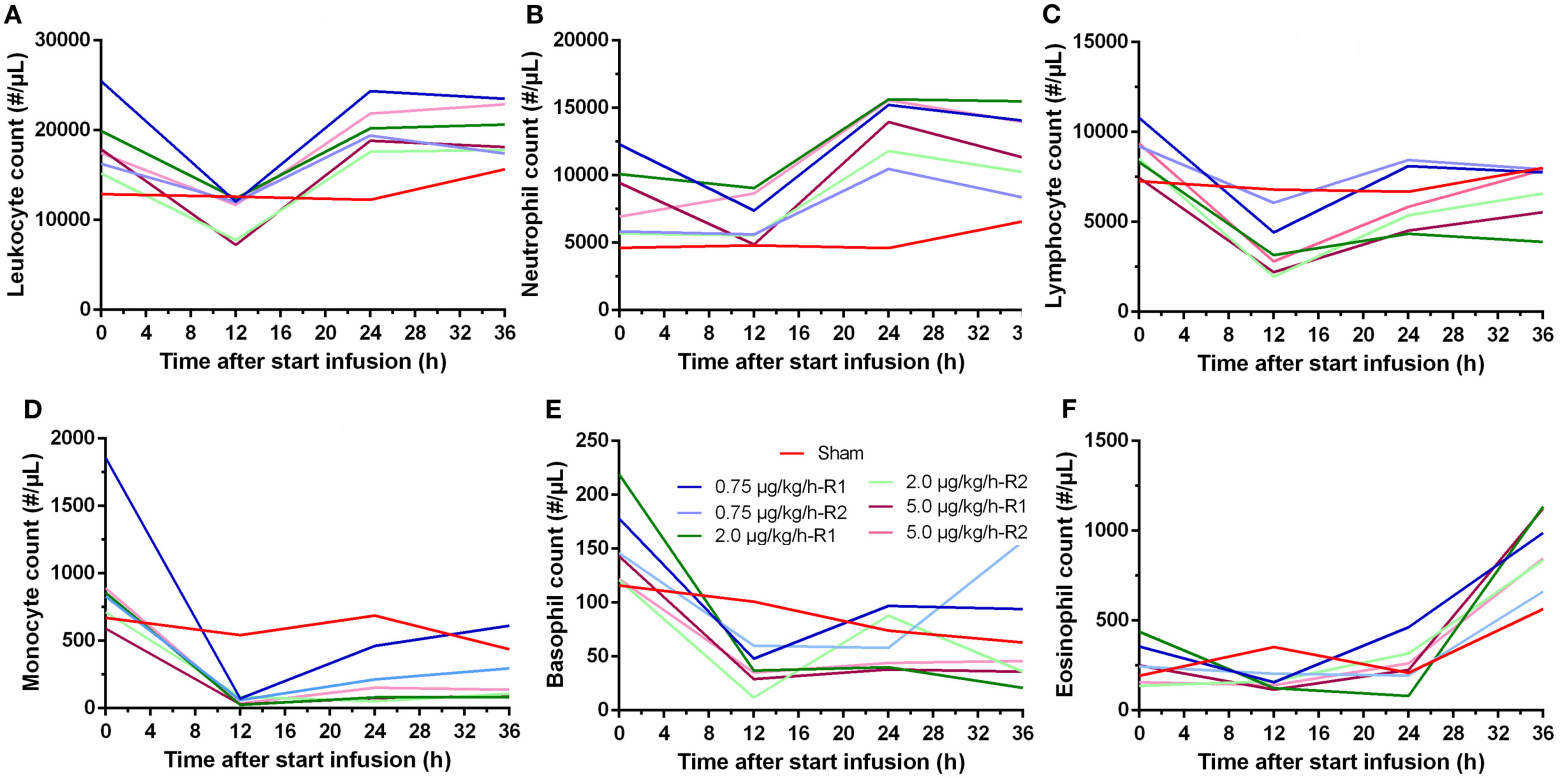
Hematological changes in leukocyte **(A)** neutrophil **(B)**, lymphocyte **(C)**, monocyte **(D)**, basophil **(E)**, and eosinophil **(F)** count over time during a 36 h continuous intravenous infusion of saline (6 ml/kg/h, sham, *n* = 1) or saline (6 ml/kg/h) in combination with LPS (0.75; 2.0, and 5.0 μg/kg/h, *n* = 2/group).

Important hematological changes were observed during the 36 h of LPS administration. In general, no pronounced differences were observed between the three LPS doses. Overall, an initial drop in leukocyte count was observed after 12 h, but recovery was present after 24 h (Figure 3A). An initial decrease in neutrophil count was observed after 12 h, which was followed by a strong increase in neutrophil count after 24 and 36 h relative to the count before the start of the LPS administration, as shown in Figure 3B. A significant decrease in lymphocyte count was noticed after the start of the LPS administration. This decrease reached its maximum after 12 h, but recovery was noticed after 24 h of LPS infusion, as illustrated in Figure 3C. Regarding the monocyte count, a pronounced decrease in monocytes was observed after 12 h with slight recovery at 24 and 36 h after the start of the LPS infusion (Figure 3D). An overall tendency of faster recovery was noticed in the piglets receiving a low dose of LPS (0.75 μg/kg/h), but this was not substantiated by statistical analysis. The basophil count decreased notably after 12 h of LPS infusion, but again, recovery was noticed after 24 h (Figure 3E). Furthermore, a marked increase in eosinophil count after 36 h of LPS administration could be observed in the LPS-treated piglets (Figure 3F), although this tendency was also observed in the sham piglet.

### Prostaglandin Plasma Concentrations

**Figure 4:**
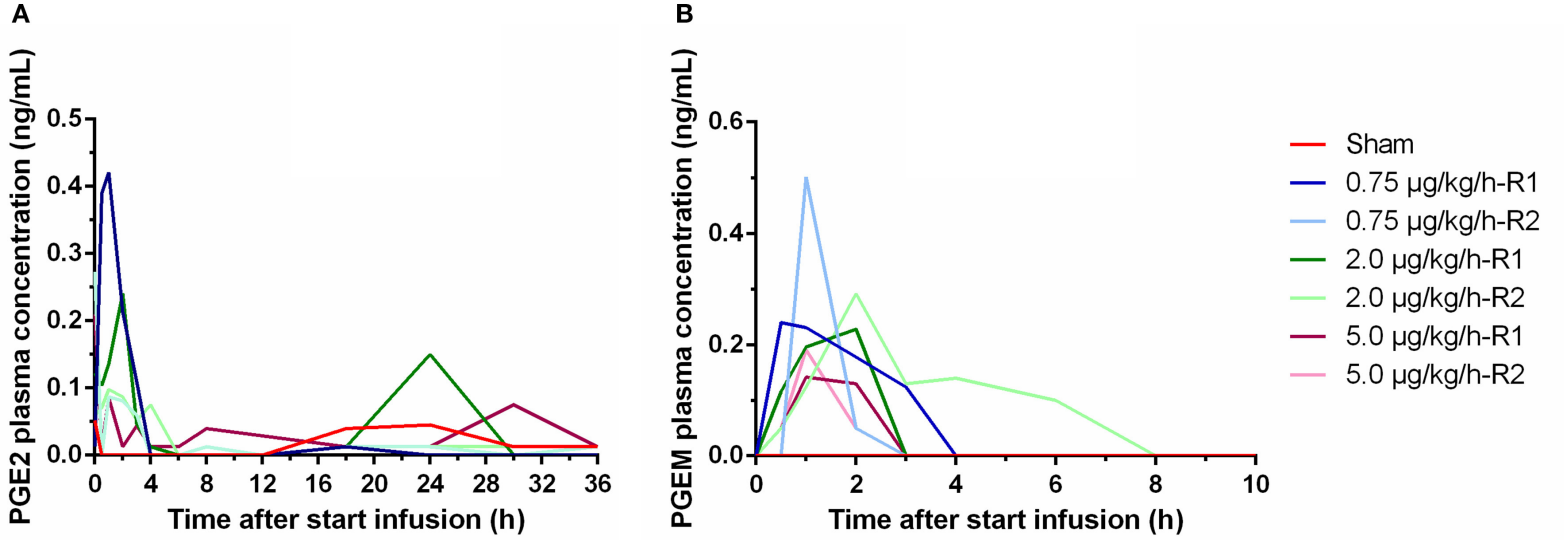
Plasma concentrations of PGE_2_ **(A)** and 13,14-dihydro-15-keto PGE2 (PGEM) **(B)** over time during a 36 h continuous intravenous infusion of either 0.9% NaCl solution (6 ml/kg/h, sham, *n* = 1) or 0.9% NaCl solution (6 ml/kg/h) in combination with LPS (0.75; 2.0, and 5.0 μg/kg/h, *n* = 2/group). Values below LOQ were depicted as LOQ/2, values below the LOD were set as 0.

In the LPS groups, plasma concentrations above the LOQ were measured for PGE_2_ and PGEM. Only in one piglet, a single quantifiable PGAM plasma concentration was observed. The PGE_2_ plasma concentrations ranged from <LOD to 0.420 ng/ml. PGEM plasma concentration ranged from <LOD to 0.502 ng/ml. Both PGE_2_ and PGEM plasma concentrations increased to reach peak value already at 1–2 h a.s.i. in all LPS receiving piglets, whereas in the sham piglet, negligible levels of PGE_2_ or PGEM were measured (Figure 4). After 4 h of LPS infusion, the PGE_2_ concentrations in the LPS group were already largely below the LOQ, and no detectable PGEM concentrations were perceived 8 h a.s.i. Due to the high inter-individual variability, no differences in the plasma concentrations of both prostaglandins could be noticed between the different LPS doses.

### Clearances of Renal Markers

To evaluate the effect of LPS on the renal function, three types of renal markers were administered simultaneously to each piglet. For one piglet (0.75 μg/kg/h-R1), the administration of the PAH-creatinine solution was only partially successful. Therefore, no clearance values for PAH and creatinine were available for that piglet after 24 h of LPS administration. For iohexol and creatinine, a two-compartmental model with first-order elimination was selected as structural PK model. For PAH, a one-compartmental model with first-order elimination best fitted the data.

**Figure 5:**
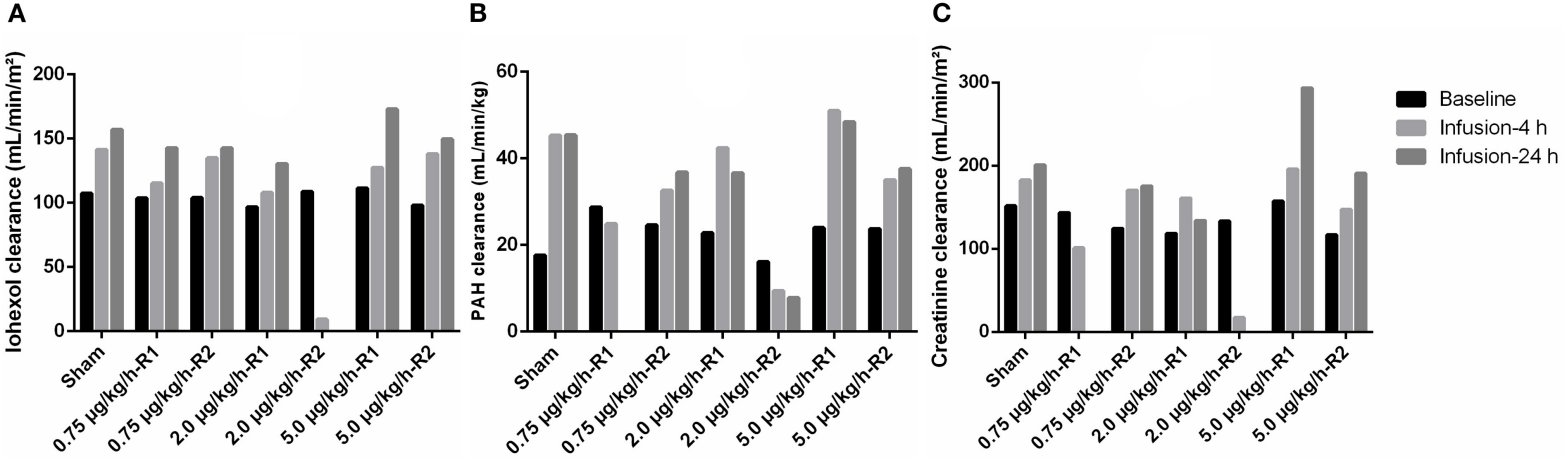
Total body clearances of the renal markers iohexol [**A**, 64.7 mg/kg body weight (BW)], para-aminohippuric acid (PAH, **B**, 10 mg/kg BW) and creatinine (**C**, 40 mg/kg BW) at baseline, and at 4 (infusion-4 h) and 24 (infusion-24 h) hours after the start of a 36 h continuous intravenous infusion of either 0.9% NaCl solution (6 ml/kg/h, sham, *n* = 1) or 0.9% NaCl solution (6 ml/kg/h) in combination with LPS (0.75; 2.0, and 5.0 μg/kg/h, *n* = 2/group). R1 and R2 represent the two replicates per dose.

As can be seen in Figure 5A, an increase in iohexol clearance, a marker of the GFR, was observed in all but one pig after 4 and 24 h of CRI infusion. This increase was more pronounced 24 h than 4 h a.s.i. Remarkable was the observation that the sham pig, only receiving a CRI of 0.9% NaCl solution, also demonstrated an enhanced iohexol clearance. A decreased renal function was observed in the piglet (replicate 2) receiving a dose of 2 μg/kg/h LPS. In this piglet, only one measurement of the GFR could be performed, since iohexol was not yet completely cleared from the body 20 h after the first iohexol administration.

Similar results were obtained for the PAH clearance, which is an indicator of the renal plasma flow. Overall, an increase in PAH clearance was observed a.s.i. In accordance with the results of iohexol, in one piglet receiving 2.0 μg/kg/h LPS, a decreased PAH clearance was observed (Figure 5B). Compared with the results of iohexol, the differences in clearance at 4 and 24 h of infusion were less pronounced.

**Figure 6:**
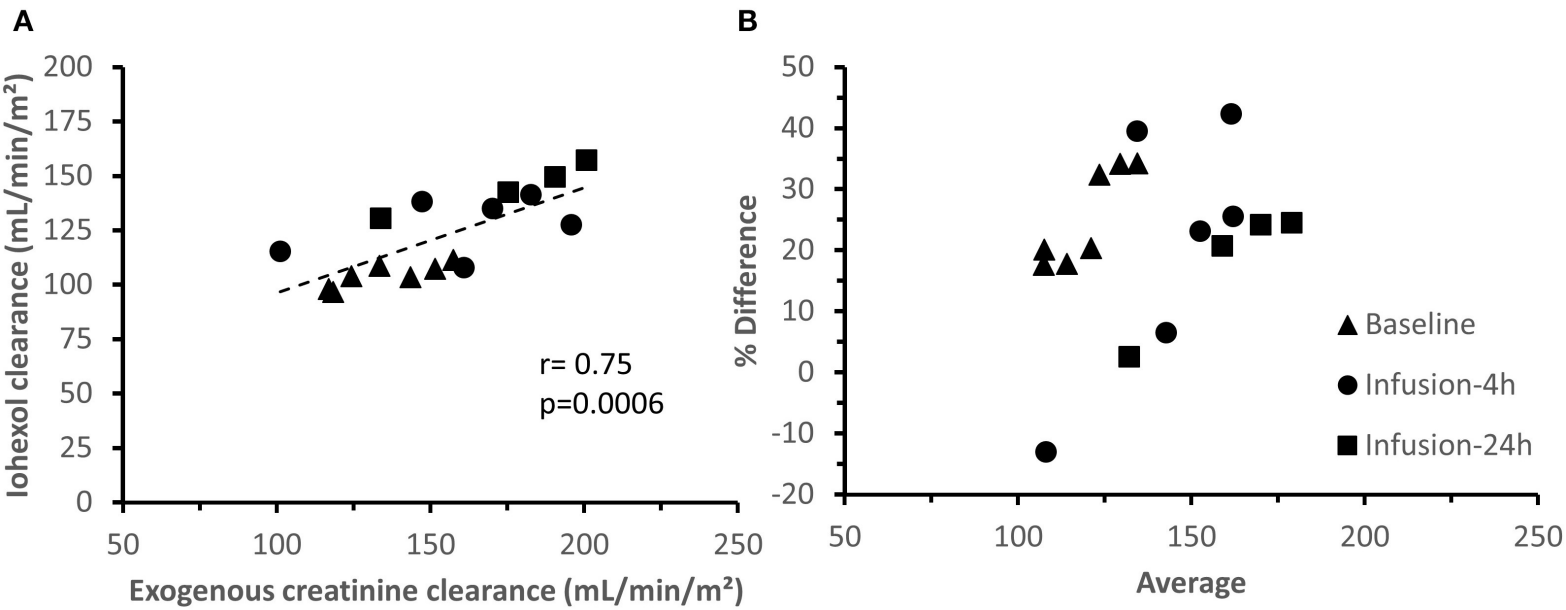
Correlation **(A)** and agreement **(B)** between iohexol and creatinine total body clearances. B presents a Bland-Altman plot of the difference between iohexol and creatinine clearance, expressed as percentage [(creatinine clearance—iohexol clearance)/average)] vs. the average of the clearances. The measurements at the distinct measuring periods are depicted with different symbols.

For exogenous creatinine, quite similar results were obtained as for iohexol (Figure 5C). A moderate, but highly significant correlation (*r* = 0.75; *p* = 0.0006) was observed between iohexol and exogenous creatinine clearances (Figure 6A). Two GFR measurements (2 μg/kg/h-R2, infusion-4 h and 5 μg/kg/h-R1, infusion-24 h) were considered as statistical outliers and therefore removed from the data set. Furthermore, the GFR calculated as exogenous creatinine clearance was, on average, 25.80 ± 16.66% higher than when calculated as iohexol clearance. This difference is illustrated in a Bland–Altman plot (Figure 6B).

### Neutrophil Gelatinase-Associated Lipocalin Plasma and Urinary Concentrations

**Figure 7:**
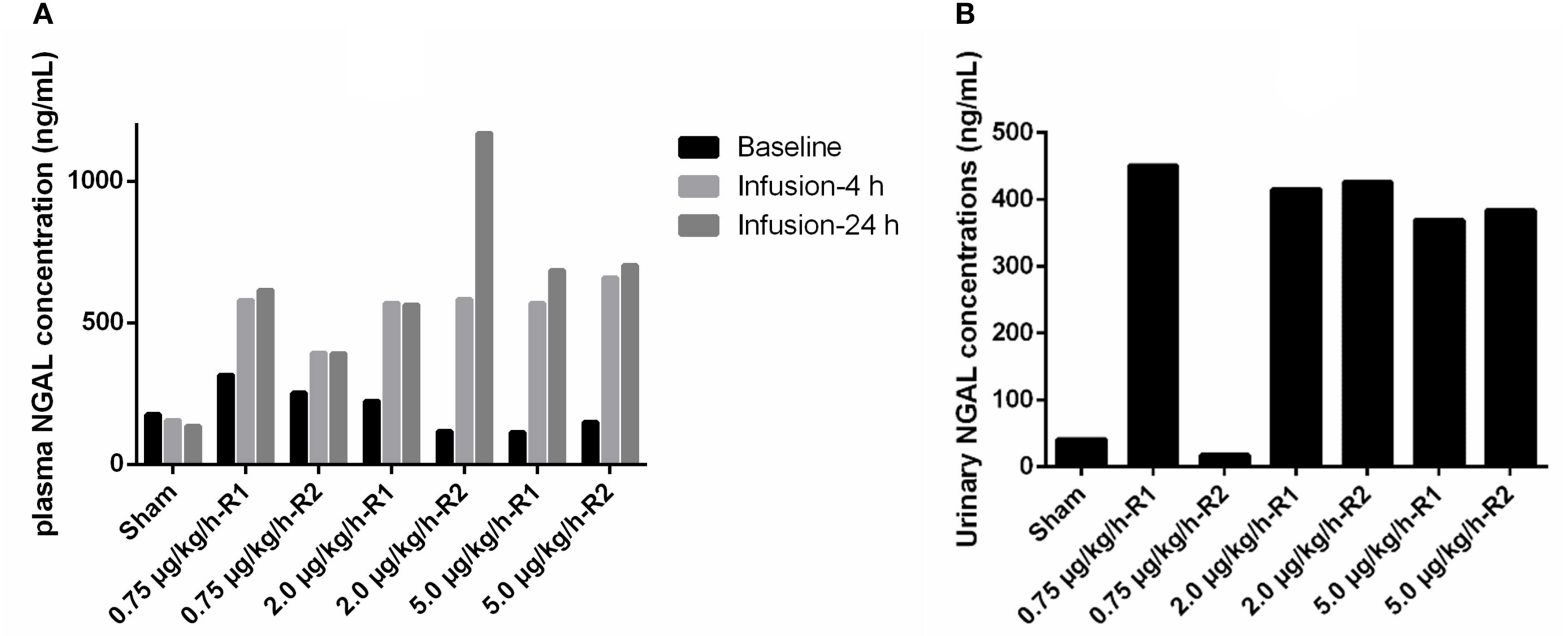
**(A)** Plasma neutrophil gelatinase-associated lipocalin (NGAL) concentrations at baseline, and at 4 and 24 h after the start of 36 h a continuous intravenous infusion of either 0.9% NaCl solution (6 ml/kg/h, sham, *n* = 1) or 0.9% NaCl solution (6 ml/kg/h) in combination with LPS (0.75; 2.0 and 5.0 μg/kg/h, *n* = 2/group). **(B)** NGAL concentrations in voided urine, collected after 4 h after the start of the respective infusions.

At baseline, pNGAL concentrations ranged from 112 to 315 ng/m in all piglets. pNGAL levels increased in all LPS-treated piglets as depicted in Figure 7A. The piglet showing a decreased renal function demonstrated the most pronounced increase in pNGAL concentrations after 24 h of LPS infusion. Additionally, pNGAL levels seemed LPS dose independent. In all but one LPS treated pig, elevated uNGAL concentrations, ranging between 369 and 451 ng/ml, were observed compared with the sham piglet (Figure 7B). Again, the LPS effect seemed dose independent.

### Morphological Parameters

**Figure 8:**
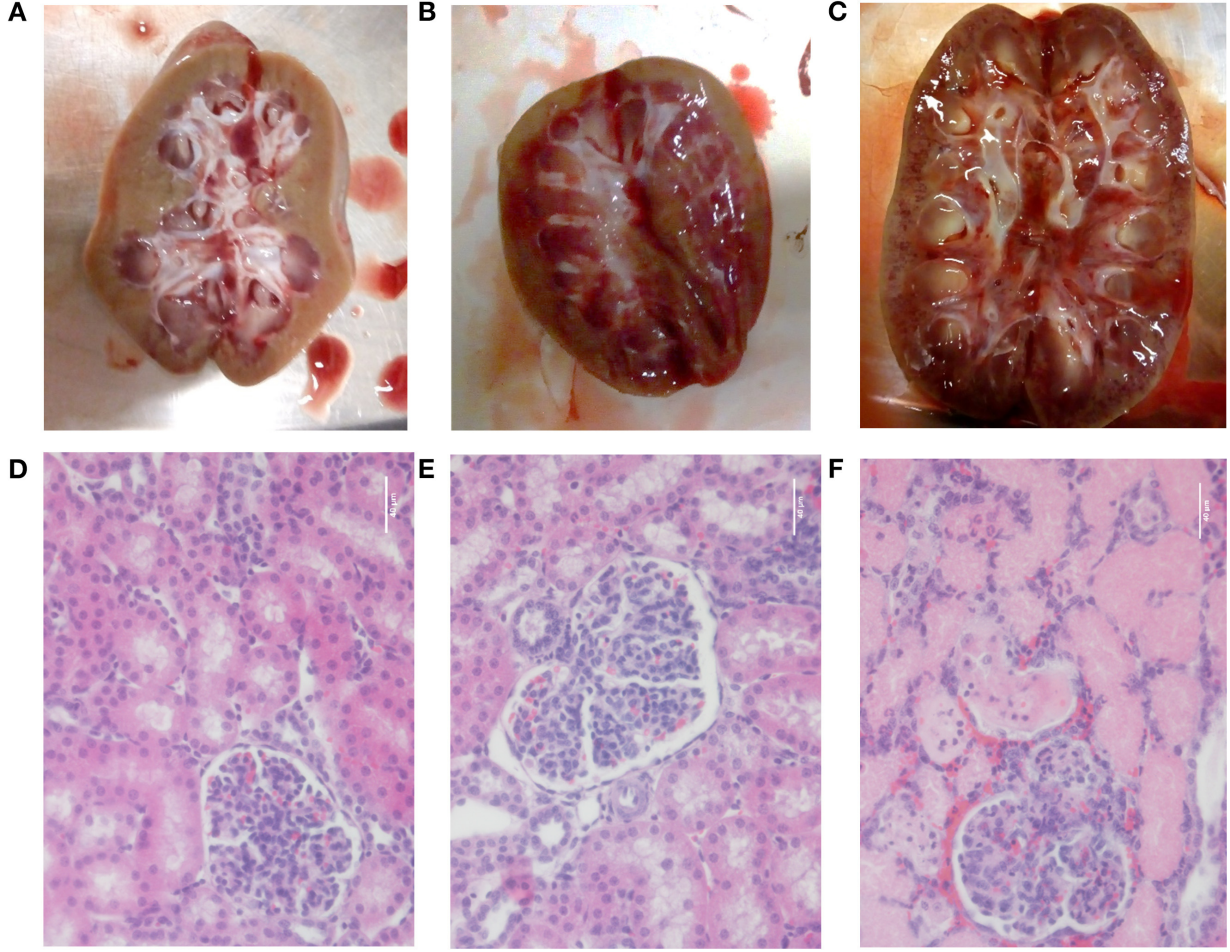
Pictures of a longitudinal section of the kidney and after H&E staining (magnification 400×) of the renal cortex of the sham pig receiving saline (6 ml/kg/h) **(A,D**), a pig receiving saline (6 ml/kg/h) in combination with a 5 μg/kg/h LPS infusion **(B,E)** and the pig demonstrating a reduced renal function after 36 h of saline infusion (6 ml/kg/h) in combination with 2 μg/kg/h LPS **(C,F)**. Scale bar represents 40 μm.

Upon necropsy, macroscopic renal abnormalities were observed in the piglet demonstrating a reduced renal function. Various multifocal petechiation areas were observed on the cross sections of both its kidneys as presented in Figure 8C. No macroscopic differences were observed between the other LPS-receiving piglets and the sham piglet (Figures 8A,B). Similar observations were made at microscopic level. Again, a clear effect of LPS infusion was observed at the level of the tubules and glomeruli in the piglet displaying a diminished renal function. Destruction of the lining epithelium of the renal tubules was observed, resulting in casts filling the lumens of the tubules Figure 8F. Furthermore, again, no differences in lesions could be observed between the other LPS receiving piglets and sham piglet Figures 8D,E.

## Discussion

The complex nature of human sepsis have contributed to the development of experimental sepsis animal models to study the human pathophysiology of sepsis (24). The piglet has proven to be very relevant as preclinical model in biochemical research due to its high degree of anatomical and physiological similarities with humans (15). The structure, function, and physiology of the mature porcine kidney is postulated to be comparable to that of humans, making pigs especially an interesting model to study renal (patho)physiology in case of sepsis (13, 14, 42). Furthermore, renal porcine maturation, including maturation of renal blood flow and GFR, development of tubulo-glomerular feedback, and response to diuretics, demonstrated similarities with the human infant (43).

In the current study, a 36-h continuous infusion with LPS was performed to elicit the clinical signs of sepsis and to study sepsis-induced changes in renal function. Conscious, non-sedated animals were used to avoid the possible confounding effects of anesthesia (44). The administered doses were selected based on literature (45–47). The three different doses were chosen in order to assess a possible dose-dependent effect of LPS on kidney function. Equally important, these doses were still low enough to allow the piglets to survive without any pulmonary or cardiovascular support. Fluid therapy was applied as this better simulates human sepsis in clinical settings. The rate of saline administration in this study was based on the daily amount of IV fluids given to critically ill children and the reported porcine daily water intake values of that age category. In the study of Van Der Heggen et al. the amount of IV fluids administered to pediatric patients admitted to the ICU ranged between 3.4 and 6 ml/kg/h (interquartile range) (25). Reported porcine daily water intake values are highly variable and ranges between 4.2 and 7.7 ml/kg/h (48–50). With a value of 6 ml/kg/h, fluid requirements of pigs are met, and the IV fluid volumes administered to pediatric ICU patients are approached. The sham piglet received saline at an equal rate, since it is postulated that fluid administration can also contribute to the development of ARC (10).

Within 1 h after the start of the LPS infusion, BT increased and stayed generally elevated during the whole experimental period. PGE_2_ is postulated to be an important mediator of fever (51). To the best of our knowledge, this is the first study examining the time course of PGE_2_ and its metabolites, PGEM, and PGAM, after continuous administration of LPS. The 36-h LPS infusion initially evoked a transient peak in both PGE_2_ and PGEM plasma levels approximately at 1 h after the start of the LPS administration, after which plasma concentrations of both compounds declined. After 3–6 h, no to negligible levels were present. These observations were similar to those reported in case LPS was administered as a single bolus injection (52–54). However, in contrast to the study of Wyns et al. (52), where quantifiable PGAM concentrations were observed up to 3 h after bolus LPS injection, most PGAM levels were below LOQ in the current study. Nevertheless, quantifiable PGEM concentrations were observed, which was not determined in the former study. Notwithstanding, PGE_2_ is considered to be a central mediator in the febrile response, the BT in the current study remained elevated even after plasma PGE_2_, and PGEM concentrations declined. This indicates that PGE_2_ plasma levels and BT a have minor direct association (55). Besides a BT elevation, the piglets demonstrated a concomitant increase in heart and respiratory rates. These latter changes were generally present till 8 h a.s.i. Endotoxin tolerance could be responsible for this observation, although this phenomenon is primarily described after repeated LPS challenge. Nonetheless, there are indications in rat and pigs that it also occurs during continuous LPS administration (22, 56).

Regarding hematological changes, similar trends in changes in white blood cell counts as observed in the current study after continuous LPS infusion are reported in other animal models after LPS administration either as a bolus injection or infusion (57–60). Generally, an initial drop occurs in counts of leukocytes, neutrophils, lymphocytes, and monocytes after (the start of) the LPS administration that is followed by recovery. Changes in leukocyte count reflect predominantly the evolution in neutrophil and lymphocyte count, since monocytes, and especially basophils and eosinophils, each account for only a minor percentage of the total leukocytes. In the present study, a small drop in neutrophil count against baseline was observed, whereafter a strong elevation in neutrophils was present at 24 h a.s.i. This observation was similar as observed in the bolus model of Williams et al. (57). In the present study, the peak observed 24 h a.s.i. was followed by a slight decrease in neutrophil count 36 h a.s.i., though levels remained above baseline measurements. For the lymphocyte and monocyte counts, a notable decrease was observed after 12 h of LPS infusion. In both cases, a recovery in count was noticed after 24 and 36 h, but the counts stayed below the initial baseline measurements. In contrast, in the porcine LPS bolus model of Williams et al. (57), a decline in circulating lymphocyte and monocyte levels was present at 5.5 h after the LPS dosing, but at 24 h, these counts rose above baseline.

Sepsis frequently causes multiple organ dysfunction (MOD) with the kidney as one of the most vulnerable organs. Several studies have shown that LPS infusion is able to cause renal dysfunction accompanied with a decrease in GFR (61–63). In this study, a concept was introduced to simulate ARC by infusion of a low to medium dose of LPS over a prolonged period of time in piglets. This ARC porcine model could contribute to the unraveling of the mechanisms underlying ARC. During the study, five out of six endotoxemic piglets showed a marked increase in GFR after LPS administration. One piglet showed a severe decrease in GFR, which can be caused by the porcine dermatitis and nephropathy syndrome (PDNS) (64). The cause of PDNS is uncertain, but it hypothesized that a type III hypersensitivity reaction provokes the disease (65). Hereby, LPS can act as an antigen in the immune complexes formed during this reaction. Typically, pathological findings of PDNS were present in this piglet, among which enlarged and pale kidneys with petechial hemorrhages (Figure 8C) and serous effusions in body cavities (Supplementary Figure 3) (64). No distinct skin lesions were observed in this piglet.

Likewise, the five piglets receiving LPS in combination with 0.9% NaCl solution, the sham piglet, only receiving 0.9% NaCl solution, displayed an elevated GFR over the time course of the study. Though the definitive causes of ARC remain an enigma, fluid resuscitation is regarded as a factor in the development of ARC. The chief theory is that increased fluid administration leads to a raise in cardiac output, thereby augmenting the circulation in the kidneys (66). However, in multiple human studies, the volumes of administered fluids and fluid balance were not considered as significant covariates for the prevalence of ARC (10, 25, 67–69). Previously, Wan et al. (70) demonstrated that extensive fluid administration over a short period of time (1 L/15 min) significantly increased creatinine clearance in sheep. More recently, it was shown that continuous fluid administration in pigs led to increases in iohexol and amikacin clearances. This illustrates the potential detrimental impact of fluid therapy on drug pharmacokinetics and drug efficacy (71).

To the authors' knowledge, the plasma exogenous creatinine clearance test has not been previously performed in pigs, in contrast to humans, birds, cats, and dogs (72–75). The exogenous creatinine clearance, which is a measure of the GFR, demonstrated an acceptable correlation with iohexol (*r* = 0.75, *p* = 0.0006), the former being, on average, 25.80% higher compared with the latter. Also in cats and birds, the plasma clearance of exogenous creatinine was higher than that of iohexol (72, 75). This discrepancy could be attributed to the fact that creatinine, besides being filtrated, is also partially tubular secreted, as reported by several in humans (73, 76, 77). Overall, it can be concluded that for the accurate estimation of the GFR, iohexol clearance seems more appropriate compared with exogenous creatinine clearance. In this study the total PAH clearance was used to estimate the renal plasma flow. Four out of six LPS-receiving piglets as well as the sham pig showed an increase in PAH clearance after the start of therapy. Since piglets are also able to metabolize PAH very efficiently, the total clearance of PAH in the current study represents both renal and metabolic clearance (14). Hence, the changes in PAH clearance could be due to changes in renal plasma flow, liver blood flow, and/or metabolizing capacity.

The intracellularly stored granule protein NGAL is released in large amounts by neutrophilic granulocytes and is a well-known biomarker for early detection of human AKI. Indeed, AKI can be observed by an increase in pNGAL and especially in uNGAL levels before changes in creatinine levels occur (78). However, recent data suggest that pNGAL levels are, to a greater extent, related to the systemic inflammatory response rather than to the pathophysiology associated with AKI (79). Consequently, pNGAL can be considered as a marker of inflammation and more specifically reflects neutrophilic granulocyte activation in plasma (79). Nevertheless, in the current exploratory study, the highest pNGAL levels after 24 h of LPS infusion were observed in the pig demonstrating a reduced kidney function. Also, in the other piglets receiving LPS, a marked increase in pNGAL concentrations was observed. Notwithstanding this intriguing novel observation, these increased pNGAL concentrations were neither reflected in a decrease in renal function, as determined by a lower GFR, nor as morphological damage at the level of the kidneys. This observation suggests that also in pigs, the increase in pNGAL concentrations is not necessarily linked to a decrease in renal function or microscopically renal damage. In addition, only a minor association between uNGAL and renal function/damage was observed. Most surprisingly, the reduced renal function and microscopical damage of piglet 2 μg/kg/h-R1 was not accompanied by a more pronounced increase in uNGAL levels when compared with the other LPS-treated piglets without renal microscopic damage. Further investigation is necessary to evaluate if the elevation of uNGAL in the LPS-treated piglets in the absence of renal microscopic damage could be an early sign of AKI development [renal stress concept, as proposed in human septic AKI (80)] or is the result of an enhanced renal elimination of extrarenally generated NGAL.

Although lack of statistical analysis due to the small sample size is a limitation of the current study, it serves as an important proof-of-concept for future experimental porcine sepsis models. With regard to the dose–response relationship between LPS and the investigated parameters, no substantial dose-dependent effects of the three investigated doses could be found. In addition, ARC was successfully induced in these studied pigs. Since the sham pig also demonstrated ARC, fluid administration may contribute to a larger extent to the development of ARC than initially thought. Therefore, it would be interesting to evaluate on a large scale the effect of fluid administration on the renal function. Furthermore, it should be investigated whether LPS administration without concomitant fluid administration would also result in ARC or would rather induce AKI.

## Data Availability Statement

The raw data supporting the conclusions of this article will be made available by the authors, without undue reservation.

## Ethics Statement

The animal study was reviewed and approved by Ethical Committee of the Faculty of Veterinary Medicine and the Faculty of Bioscience Engineering of Ghent University.

## Author Contributions

LD, SC, PDP, and MD conceived and designed the study. LD performed the experiments. LD, WV, and MD performed the data analysis. LD wrote the first draft of the manuscript. All authors critically reviewed several drafts of the manuscript.

## Conflict of Interest

The authors declare that the research was conducted in the absence of any commercial or financial relationships that could be construed as a potential conflict of interest.

## References

[1] Fleischmann C Scherag A Adhikari NKJ Hartog CS Tsaganos T Schlattmann P. Assessment of global incidence and mortality of hospital-treated sepsis current estimates and limitations. Am J Respir Crit Care Med. (2016) 193:259–72. 10.1164/rccm.201504-0781OC 26414292

[2] Fleischmann-Struzek C Goldfarb DM Schlattmann P Schlapbach LJ Reinhart K Kissoon N. The global burden of paediatric and neonatal sepsis: a systematic review. Lancet Respir Med. (2018) 6:223–30. 10.1016/S2213-2600(18)30063-8 29508706

[3] Carcillo JA Doughty L Kofos D Frye RF Kaplan SS Sasser H. Cytochrome P450 mediated-drug metabolism is reduced in children with sepsis-induced multiple organ failure. Intensive Care Med. (2003) 29:980–4. 10.1007/s00134-003-1758-3 12698250

[4] De Cock PAJG Standing JF Barker CIS De Jaeger A Dhont E Carlier M. Augmented renal clearance implies a need for increased amoxicillin-clavulanic acid dosing in critically ill children. Antimicrob Agents Chemother. (2015) 59:7027–35. 10.1128/AAC.01368-15 26349821 PMC4604416

[5] Ince I De Wildt SN Peeters MYM Murry DJ Tibboel D Danhof M. Critical illness is a major determinant of midazolam clearance in children aged 1 month to 17 years. Ther Drug Monit. (2012) 34:381–9. 10.1097/FTD.0b013e31825a4c3a 22660604

[6] Smith BS Yogaratnam D Levasseur-Franklin KE Forni A Fong J. Introduction to drug pharmacokinetics in the critically ill patient. Chest. (2012) 141:1327–36. 10.1378/chest.11-1396 22553267

[7] Van Den Anker JN Knibbe CAJ Tibboel D. Augmented renal clearance in critically Ill pediatric patients: does it impact the outcome of pharmacotherapy?*. Pediatr Crit Care Med. (2017) 18:901–2. 10.1097/PCC.0000000000001264 28863094

[8] Baptista JP Udy AA Sousa E Pimentel J Wang L Roberts JA. A comparison of estimates of glomerular filtration in critically ill patients with augmented renal clearance. Crit Care. (2011) 15:R139 1–8. 10.1186/cc10262 21651804 PMC3219011

[9] Udy AA Roberts JA Lipman J. Implications of augmented renal clearance in critically ill patients. Nat Rev Nephrol. (2011) 7:539–43. 10.1038/nrneph.2011.92 21769107

[10] Udy AA Roberts JA Boots RJ Paterson DL Lipman J. Augmented renal clearance: implications for antibacterial dosing in the critically Ill. Clin Pharmacokinet. (2010) 49:1–16. 10.2165/11318140-000000000-00000 20000886

[11] Avedissian SN Bradley E Zhang D Bradley JS Nazer LH Tran TM. Augmented renal clearance using population-based pharmacokinetic modeling in critically Ill pediatric patients*. Pediatr Crit Care Med. (2017) 18:e388–94. 10.1097/PCC.0000000000001228 28640009

[12] Deitch EA. Animal models of sepsis and shock: a review and lessons learned. Shock. (1998) 9:1–11. 10.1097/00024382-199801000-00001 9466467

[13] Gasthuys E Vandecasteele T De Bruyne P Vande Walle J De Backer P Cornillie P. The potential use of piglets as human pediatric surrogate for preclinical pharmacokinetic and pharmacodynamic drug testing. Curr Pharm Des. (2016) 22:4069–85. 10.2174/1381612822666160303111031 26935702

[14] Dhondt L Croubels S De Paepe P Wallis SC Pandey S Roberts JA. Conventional pig as animal model for human renal drug excretion processes: unravelling the porcine renal function by use of a cocktail of exogenous markers. Front Pharmacol. (2020) 11:833. 10.3389/fphar.2020.00883 32595506 PMC7303324

[15] Swindle MM Makin A Herron AJ Clubb FJ Frazier KS. Swine as models in biomedical research and toxicology testing. Vet Pathol. (2012) 49:344–56. 10.1177/0300985811402846 21441112

[16] Fink MP Heard SO. Laboratory models of sepsis and septic shock. J Surg Res. (1990) 49:186–96. 10.1016/0022-4804(90)90260-9 2199735

[17] Wang L Hou Y Yi D Li Y Ding B Zhu H. Dietary supplementation with glutamate precursor α-ketoglutarate attenuates lipopolysaccharide-induced liver injury in young pigs. Amino Acids. (2015) 47:1309–18. 10.1007/s00726-015-1966-5 25795418

[18] Tuchscherer M Kanitz E Puppe B Tuchscherer A Stabenow B. Effects of postnatal social isolation on hormonal and immune responses of pigs to an acute endotoxin challenge. Physiol Behav. (2004) 82:503–11. 10.1016/j.physbeh.2004.04.056 15276816

[19] Burdick Sanchez NC Carroll JA Broadway PR Bass BE Frank JW. Modulation of the acute phase response following a lipopolysaccharide challenge in pigs supplemented with an all-natural *Saccharomyces cerevisiae* fermentation product. Livest Sci. (2018) 208:1–4. 10.1016/j.livsci.2017.11.022

[20] Wyns H Plessers E De Backer P Meyer E Croubels S. *In vivo* porcine lipopolysaccharide inflammation models to study immunomodulation of drugs. Vet Immunol Immunopathol. (2015) 166:58–69. 10.1016/j.vetimm.2015.06.001 26099806

[21] Guthrie G Tackett B Stoll B Martin C Olutoye O Burrin DG. Phytosterols synergize with endotoxin to augment inflammation in kupffer cells but alone have limited direct effect on hepatocytes. J Parenter Enter Nutr. (2018) 42:37–48. 10.1177/0148607117722752 28792854 PMC8279067

[22] Castegren M Skorup P Lipcsey M Larsson A Sjölin J. Endotoxin tolerance variation over 24 h during porcine endotoxemia: association with changes in circulation and organ dysfunction. PLoS ONE. (2013) 8:e53221. 10.1371/journal.pone.0053221 23326400 PMC3542331

[23] D'Orio V Wahlen C Rodriguez LM Fossion A Juchmes J Halleux J. A comparison of *Escherichia coli* endotoxin single bolus injection with low-dose endotoxin infusion on pulmonary and systemic vascular changes. Circ Shock. (1987) 21:207–16. 3552282

[24] Buras JA Holzmann B Sitkovsky M. Model organisms: animal models of sepsis: setting the stage. Nat Rev Drug Discov. (2005) 4:854–65. 10.1038/nrd1854 16224456

[25] Van Der Heggen T Dhont E Peperstraete H Delanghe JR Vande Walle J De Paepe P. Augmented renal clearance: a common condition in critically ill children. Pediatr Nephrol. (2019) 34:1099–106. 10.1007/s00467-019-04205-x 30778827

[26] Soto K Papoila AL Coelho S Bennett M Ma Q Rodrigues B. Plasma NGAL for the diagnosis of AKI in patients admitted from the emergency department setting. Clin J Am Soc Nephrol. (2013) 8:2053–63. 10.2215/CJN.12181212 24009223 PMC3848412

[27] Hjortrup PB Haase N Wetterslev M Perner A. Clinical review: predictive value of neutrophil gelatinase-associated lipocalin for acute kidney injury in intensive care patients. Crit Care. (2013) 17:211. 10.1186/cc11855 23680259 PMC3672520

[28] Cruz DN De Cal M Garzotto F Perazella MA Lentini P Corradi V. Plasma neutrophil gelatinase-associated lipocalin is an early biomarker for acute kidney injury in an adult ICU population. Intensive Care Med. (2010) 36:444–51. 10.1007/s00134-009-1711-1 19956925 PMC2820221

[29] Schlondorff D Ardaillou R. Prostaglandins and other arachidonic acis metabolites in the kidney. Kidney Int. (1986) 29:108–19. 10.1038/ki.1986.13 3083150

[30] Haupt W Fritzsche H Hohenberger W Zirngibl H. Selective cytokine release induced by serum and separated plasma from septic patients. Eur J Surg. (1996) 162:769–76. 8934105

[31] Astiz M Saha D Lustbader D Lin R Rackow E. Monocyte response to bacterial toxins, expression of cell surface receptors, and release of anti-inflammatory cytokines during sepsis. J Lab Clin Med. (1996) 128:594–600. 10.1016/S0022-2143(96)90132-8 8960643

[32] Rochwerg B Alhazzani W Sindi A Heels-Ansdell D Thabane L Fox-Robichaud A. Fluid resuscitation in sepsis: a systematic review and network meta-analysis. Ann Intern Med. (2014) 161:347–55. 10.7326/M14-0178 25047428

[33] Cook AM Hatton-Kolpek J. Augmented renal clearance. Pharmacother J Hum Pharmacol Drug Ther. (2019) 39:346–54. 10.1002/phar.2231 30723936

[34] European Commission. Directive 2010/63/EU of the European Parliament and of the council of 22 September 2010 on the protection of animals used for scientific purposes. Off J Eur Union. (2010) L276:33–79.

[35] Anonymous. Belgian Royal Decree of 29 May 2013 on the protection of animals used for scientific purposes. Belg Staatsbl. (2013) 42808–912.

[36] Gasthuys F De Boever S Schauvliege S Reyns T Levet T Cornillie P. Transsplenic portal catheterization combined with a jugular double-lumen catheter for pharmacokinetic and presystemic metabolization studies in pigs. J Vet Pharmacol Ther. (2009) 32:137–45. 10.1111/j.1365-2885.2008.01012.x 19290943

[37] Gasthuys E Schauvliege S van Bergen T Millecam J Cerasoli I Martens A. Repetitive urine and blood sampling in neonatal and weaned piglets for pharmacokinetic and pharmacodynamic modelling in drug discovery: a pilot study. Lab Anim. (2017) 51:498–508. 10.1177/0023677217692372 28178895

[38] Pelligand L House AK Summers BA Hatzis A Tivers M Elliott J. Development and validation of a tissue cage model of acute inflammation in the cat. J Vet Pharmacol Ther. (2012) 35:239–48. 10.1111/j.1365-2885.2011.01308.x 21781136

[39] Dhondt L Croubels S De Cock P De Paepe P De Baere S Devreese M. Development and validation of an ultra-high performance liquid chromatography–tandem mass spectrometry method for the simultaneous determination of iohexol, p-aminohippuric acid and creatinine in porcine and broiler chicken plasma. J Chromatogr B Anal Technol Biomed Life Sci. (2019) 1117:77–85. 10.1016/j.jchromb.2019.04.017 31004849

[40] Granström E Hamberg M Hansson G Kindahl H. Chemical instability of 15-keto-13,14-dihydro-PGE2: The reason for low assay reliability. Prostaglandins. (1980) 19:933–57. 10.1016/0090-6980(80)90127-6 7384561

[41] Fitzpatrick FA Aguirre R Pike JE Lincoln FH. The stability of 13,14-dihydro-15 keto-PGE2. Prostaglandins. (1980) 19:917–31. 10.1016/0090-6980(80)90126-4 7384560

[42] Dalmose AL Hvistendahl JJ Olsen LH Eskild-Jensen A Djurhuus JC Swindle MM. Surgically induced urologic models in swine. J Investig Surg. (2000) 13:133–145. 10.1080/08941930050075829 10933109

[43] Calvert DA Terris JM. Evaluation of the mechanism of cyclosporine A nephrotoxicity. In: Tumbleson ME Schook LB editors. Advances in Swine in Biomedical Research. New York, NY: Plenum Press (1996).

[44] Deutsch S. Effects of anesthetics on the kidney. Surg Clin North Am. (1975) 55:775–86. 10.1016/S0039-6109(16)40680-8 1166370

[45] Murphey ED Traber DL. Cardiopulmonary and splanchnic blood flow during 48 hours of a continuous infusion of endotoxin in conscious pigs: a model of hyperdynamic shock. Shock. (2000) 13:224–9. 10.1097/00024382-200003000-00009 10718380

[46] Orellana RA O'Connor PMJ Nguyen H V. Bush JA Suryawan A Carole Thivierge M. Endotoxemia reduces skeletal muscle protein synthesis in neonates. Am J Physiol - Endocrinol Metab. (2002) 283:E909–16. 10.1152/ajpendo.00220.2002 12376317

[47] Sølling C Nygaard U Christensen AT Wogensen L Krog J Tønnesen EK. Lymphocyte apoptosis is resistant to erythropoietin in porcine endotoxemia. APMIS. (2011) 119:143–54. 10.1111/j.1600-0463.2010.02704.x 21208282

[48] Bigelow JA Houpt TR. Feeding and drinking patterns in young pigs. Physiol Behav. (1988) 43:99–109. 10.1016/0031-9384(88)90104-7 3413258

[49] Brooks PH Russell SJ Carpenter JL. Water intake of weaned piglets from three to seven weeks old. Vet Rec. (1984) 115:513–5. 10.1136/vr.115.20.513 6542717

[50] SCS Boehringer Ingelheim Comm.V. Zakboek varkens: Feiten - Data - Cijfers (2018).

[51] Sehic E Székely M Ungar AL Oladehin A Blatteis CM. Hypothalamic prostaglandin E2 during lipopolysaccharide-induced fever in guinea pigs. Brain Res Bull. (1996) 39:391–9. 10.1016/0361-9230(96)00037-8 9138749

[52] Wyns H Meyer E Plessers E Watteyn A van Bergen T Schauvliege S. Modulation by gamithromycin and ketoprofen of *in vitro* and *in vivo* porcine lipopolysaccharide-induced inflammation. Vet Immunol Immunopathol. (2015) 168:211–22. 10.1016/j.vetimm.2015.09.014 26547885

[53] Peters SM Yancy H Deaver C Jones YL Kenyon E Chiesa OA. *In vivo* characterization of inflammatory biomarkers in swine and the impact of flunixin meglumine administration. Vet Immunol Immunopathol. (2012) 148:236–42. 10.1016/j.vetimm.2012.05.001 22648045

[54] Wright KJ Balaji R Hill CM Dritz SS Knoppel EL Minton JE. Integrated adrenal, somatotropic, and immune responses of growing pigs to treatment with lipopolysaccharide. J Anim Sci. (2000) 78:1892. 10.2527/2000.7871892x 10907832

[55] Engström L Ruud J Eskilsson A Larsson A Mackerlova L Kugelberg U. Lipopolysaccharide-induced fever depends on prostaglandin E2 production specifically in brain endothelial cells. Endocrinology. (2012) 153:4849–61. 10.1210/en.2012-1375 22872578

[56] Sanchez Cantu L Rode HN Christou N V. Endotoxin tolerance is associated with reduced secretion of tumor necrosis factor. Arch Surg. (1989) 124:1432–6. 10.1001/archsurg.1989.01410120082016 2589967

[57] Williams PN Collier CT Carroll JA Welsh TH Laurenz JC. Temporal pattern and effect of sex on lipopolysaccharide-induced stress hormone and cytokine response in pigs. Domest Anim Endocrinol. (2009) 37:139–47. 10.1016/j.domaniend.2009.04.004 19523782

[58] Yu DH Kim B Park J. Pathophysiologic and immunologic changes in a canine endotoxemia over a period of 24 hours. J Vet Med Sci. (2012) 74:537–44. 10.1292/jvms.11-0321 22146337

[59] Gundersen RY Ruud TE Jørgensen PF Scholz T Reinholt FP Wang JE. Systemic administration of enamel matrix derivative to lipopolysaccharide- challenged pigs: effects on the inflammatory response. Surg Infect. (2008) 9:161–9. 10.1089/sur.2007.007 18426348

[60] Kluess J Kahlert S Panther P Diesing AK Nossol C Rothkötter HJ. Systemic *E. coli* lipopolysaccharide but not deoxynivalenol results in transient leukopenia and diminished metabolic activity of peripheral blood mononuclear cells *ex vivo*. Mycotoxin Res. (2015) 31:41–50. 10.1007/s12550-014-0212-4 25315977

[61] Castellano G Stasi A Intini A Gigante M Di Palma AM Divella C. Endothelial dysfunction and renal fibrosis in endotoxemia-induced oliguric kidney injury: possible role of LPS-binding protein. Crit Care. (2014) 18:520. 10.1186/s13054-014-0520-2 25261195 PMC4205288

[62] Sølling C Christensen AT Nygaard U Krag S Frøkiær J Wogensen L. Erythropoietin does not attenuate renal dysfunction or inflammation in a porcine model of endotoxemia. Acta Anaesthesiol Scand. (2011) 55:411–21. 10.1111/j.1399-6576.2011.02396.x 21342148

[63] Fenhammar J Andersson A Forestier J Weitzberg E Sollevi A Hjelmqvist H. Endothelin receptor a antagonism attenuates renal medullary blood flow impairment in endotoxemic pigs. PLoS ONE. (2011) 6:e21534. 10.1371/journal.pone.0021534 21760895 PMC3132177

[64] Drolet R Thibault S D'Allaire S Thomson JR Done SH. Porcine dermatitis and nephropathy syndrome (PDNS): An overview of the disease. J Swine Heal Prod. (1999) 7:283–5.

[65] Wellenberg GJ Stockhofe-Zurwieden N De Jong MF Boersma WJA Elbers ARW. Excessive porcine circovirus type 2 antibody titres may trigger the development of porcine dermatitis and nephropathy syndrome: a case-control study. Vet Microbiol. (2004) 99:203–14. 10.1016/j.vetmic.2004.01.001 15066723

[66] Chen IH Nicolau DP. Augmented renal clearance and how to augment antibiotic dosing. Antibiotics. (2020) 9:1–12. 10.3390/antibiotics9070393 32659898 PMC7399877

[67] Campassi ML Gonzalez MC Masevicius FD Vazquez AR Moseinco M Navarro NC. Incremento da depuração renal em pacientes gravemente enfermos: Incidência, fatores associados e efeitos no tratamento com vancomicina. Rev Bras Ter Intensiva. (2014) 26:13–20. 10.5935/0103-507X.20140003 24770684 PMC4031886

[68] Hirai K Ishii H Shimoshikiryo T Shimomura T Tsuji D Inoue K. Augmented renal clearance in patients with febrile neutropenia is associated with increased risk for subtherapeutic concentrations of vancomycin. Ther Drug Monit. (2016) 38:706–10. 10.1097/FTD.0000000000000346 27681114

[69] Declercq P Nijs S D'Hoore A Van Wijngaerden E Wolthuis A De Buck Van Overstraeten A. Augmented renal clearance in non-critically ill abdominal and trauma surgery patients is an underestimated phenomenon: a point prevalence study. J Trauma Acute Care Surg. (2016) 81:468–77. 10.1097/TA.0000000000001138 27257707

[70] Wan L Bellomo R May CN. The effects of normal and hypertonic saline on regional blood flow and oxygen delivery. Anesth Analg. (2007) 105:141–7. 10.1213/01.ane.0000266438.90360.62 17578969

[71] Dhondt L Croubels S De Paepe P Goethals K De Cock P Devreese M. Unraveling the contribution of fluid therapy to the development of augmented renal clearance in a piglet model. (2021) 11:607101. 10.3389/fphar.2020.607101 33574754 PMC7870502

[72] Gasthuys E Montesinos A Caekebeke N Devreese M De Baere S Ardiaca M. Comparative physiology of glomerular filtration rate by plasma clearance of exogenous creatinine and exo-iohexol in six different avian species. Sci Rep. (2019) 9:19699. 10.1038/s41598-019-56096-5 31873143 PMC6928228

[73] Miller BF Winkler AW. The renal excretion of endogenous creatinine in man. Comparison with exogenous creatinine and inulin. J Clin Invest. (1938) 17:31–40. 10.1172/JCI100925 16694545 PMC424940

[74] Watson ADJ Lefebvre HP Concordet D Laroute V Ferré JP Braun JP. Plasma exogenous creatinine clearance test in dogs: comparison with other methods and proposed limited sampling strategy. J Vet Intern Med. (2002) 16:22. 10.1111/j.1939-1676.2002.tb01603.x 11826881

[75] van Hoek IM Lefebvre HP Paepe D Croubels S Biourge V Daminet S. Comparison of plasma clearance of exogenous creatinine, exo-iohexol, and endo-iohexol over a range of glomerular filtration rates expected in cats. J Feline Med Surg. (2009) 11:1028–30. 10.1016/j.jfms.2009.07.005 19679501 PMC11318765

[76] Bauer JH Brooks CS Burch RN. Clinical appraisal of creatinine clearance as a measurement of glomerular filtration rate. Am J Kidney Dis. (1982) 2:337–46. 10.1016/S0272-6386(82)80091-7 7148824

[77] Kim KE Onesti G Ramirez O Brest AN Swartz C. Creatinine clearance in renal disease. A Reappraisal. Br Med J. (1969) 4:11–4. 10.1136/bmj.4.5674.11 5822077 PMC1629614

[78] Mårtensson J Bell M Oldner A Xu S Venge P Martling CR. Neutrophil gelatinase-associated lipocalin in adult septic patients with and without acute kidney injury. Intensive Care Med. (2010) 36:1333–40. 10.1007/s00134-010-1887-4 20397003

[79] Söderberg E Eriksson M Larsson A Lipcsey M. The impact of hydrocortisone treatment on neutrophil gelatinase-associated lipocalin release in porcine endotoxemic shock. Intensive Care Med Exp. (2017) 5:4. 10.1186/s40635-017-0117-6 28101752 PMC5243238

[80] De Loor J Decruyenaere J Demeyere K Nuytinck L Hoste EAJ Meyer E. Urinary chitinase 3-like protein 1 for early diagnosis of acute kidney injury: a prospective cohort study in adult critically ill patients. Crit Care. (2016) 20:38. 10.1186/s13054-016-1192-x 26864834 PMC4750195

