# Supplementary material for: The Development of a Juvenile Porcine Augmented Renal Clearance Model Through Continuous Infusion of Lipopolysaccharides: An Exploratory Study
Source: Front Vet Sci. 2021 Apr 29;8:639771. doi: 10.3389/fvets.2021.639771 (PMC8116505; doi:10.3389/fvets.2021.639771)
Supplement: Supplementary file 1 [file Data_Sheet_1.docx]

**The development of a juvenile porcine augmented renal clearance model THROUGH CONTINUOUS INFUSION OF lipopolysaccharides: AN exploratory study**

Laura Dhondt^1^, Siska Croubels^1^, Pieter De Cock^2,3,4^, Evelyne Meyer^1^, Wim Van Den Broeck^5^, Peter De Paepe^3^, Mathias Devreese^1*^

^1^ Department of Pharmacology, Toxicology and Biochemistry, Salisburylaan 133, Ghent University, Merelbeke, Belgium

^2^ Department of Pharmacy, Ghent University Hospital, Corneel Heymanslaan 10, Ghent, Belgium

^3^ Heymans Institute of Pharmacology, Ghent University, Corneel Heymanslaan 10, Ghent, Belgium

^4^ Department of Paediatric Intensive Care, Ghent University Hospital, Corneel Heymanslaan 10, Ghent, Belgium

^5^ Department of Morphology, Faculty of Veterinary Medicine, Ghent University, Salisburylaan 133, Merelbeke, Belgium

*Corresponding author. E-mail address: Mathias.Devreese@UGent.be

**Supplementary material
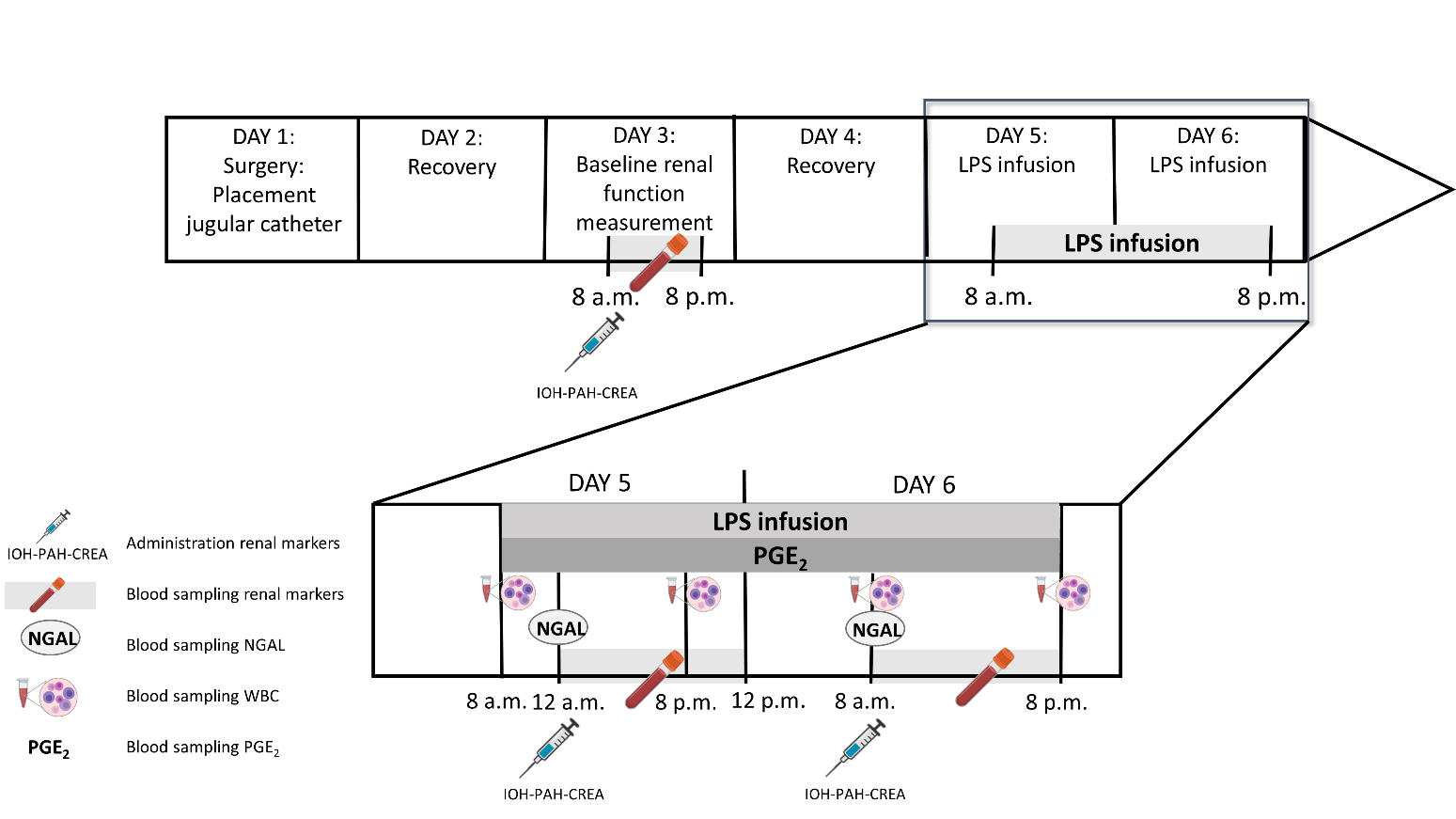
**

PGE_2_= prostaglandin E_2_; WBC= white blood cell count; NGAL= neutrophil gelatinase-associated lipocalin; IOH= iohexol; PAH= para-aminohippuric acid; CREA= creatinine; LPS= lipopolysaccharide

**Figure S1:** Graphical illustration of the trial design.

**
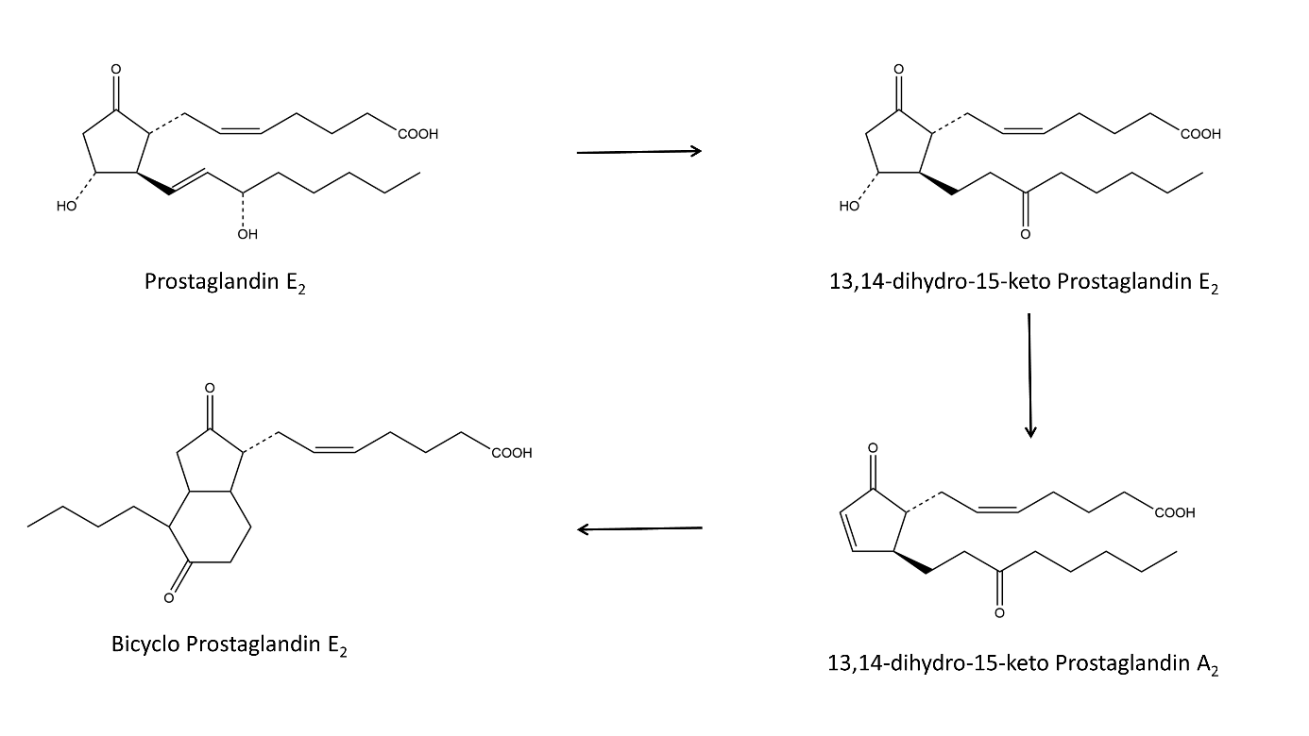
**

**Figure S2:** Conversion of prostaglandin E_2_ to bicyclo- prostaglandin E_2_.


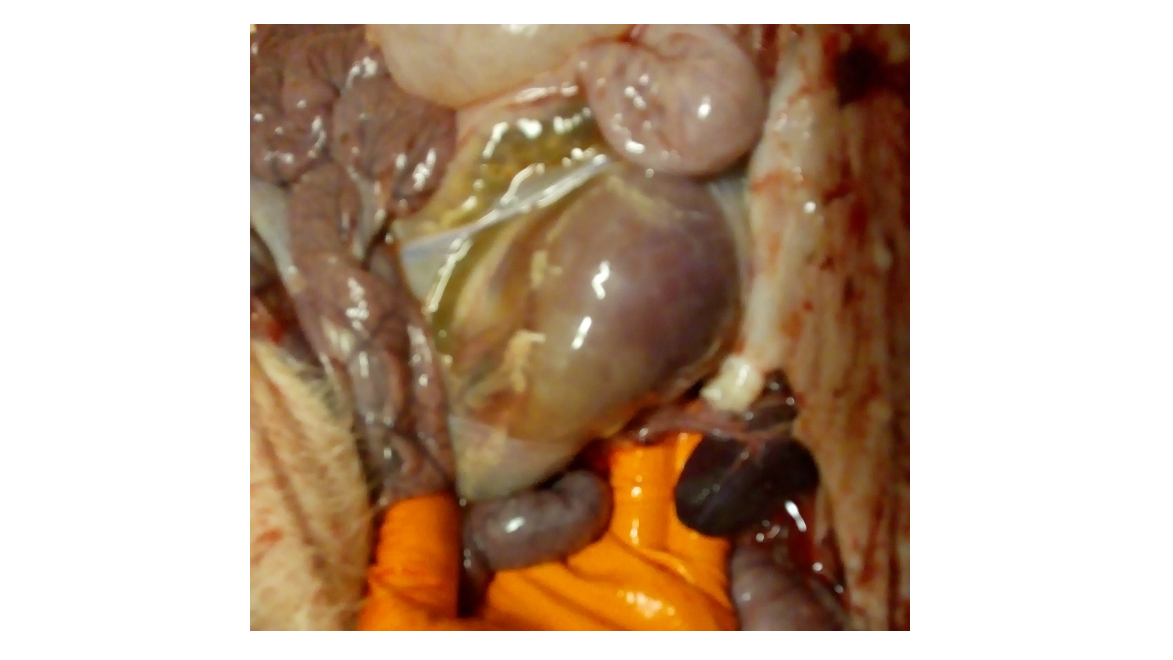


**Figure S3**: Body cavity of the piglet demonstrating a reduced renal function after 36 hours of saline infusion (6 mL/kg/h) in combination with LPS at a rate of 2 µg/kg/h. Serous effusions are present.
